# Macrostomum lignano as a model to study the genetics and genomics of parasitic flatworms

**DOI:** 10.18699/VJ21.013

**Published:** 2021-02

**Authors:** K.V. Ustyantsev, V.Yu. Vavilova, A.G. Blinov, E.V. Berezikov

**Affiliations:** Institute of Cytology and Genetics of Siberian Branch of the Russian Academy of Sciences, Novosibirsk, Russia; Institute of Cytology and Genetics of Siberian Branch of the Russian Academy of Sciences, Novosibirsk, Russia; Institute of Cytology and Genetics of Siberian Branch of the Russian Academy of Sciences, Novosibirsk, Russia; Institute of Cytology and Genetics of Siberian Branch of the Russian Academy of Sciences, Novosibirsk, Russia

**Keywords:** flatworms, parasitic flatworms, model organism, плоские черви, паразитические черви, модельный организм

## Abstract

Hundreds of millions of people worldwide are infected by various species of parasitic flatworms. Without treatment, acute and chronical infections frequently lead to the development of severe pathologies and even death. Emerging data on a decreasing efficiency of some important anthelmintic compounds and the emergence of resistance to them force the search for alternative drugs. Parasitic flatworms have complex life cycles, are laborious and expensive in culturing, and have a range of anatomic and physiological adaptations that complicate the application of standard molecular-biological methods. On the other hand, free-living flatworm species, evolutionarily close to parasitic flatworms, do not have the abovementioned difficulties, which makes them potential alternative models to search for and study homologous genes. In this review, we describe the use of the basal free-living flatworm Macrostomum lignano as such a model. M. lignano has a number of convenient biological and experimental properties, such as fast reproduction, easy and non-expensive laboratory culturing, optical body transparency, obligatory sexual reproduction, annotated genome and transcriptome assemblies, and the availability of modern molecular methods, including transgenesis, gene knockdown by RNA interference, and in situ hybridization. All this makes M. lignano amenable to the most modern approaches of forward and reverse genetics, such as transposon insertional mutagenesis and methods of targeted genome editing by the CRISPR/Cas9 system. Due to the availability of an increasing number of genome and transcriptome assemblies of different parasitic flatworm species, new knowledge generated by studying M. lignano can be easily translated to parasitic flatworms with the help of modern bioinformatic methods of comparative genomics and transcriptomics. In support of this, we provide the results of our bioinformatics search and analysis of genes homologous between M. lignano and parasitic flatworms, which predicts a list of promising gene targets for subsequent research.

## Abstract (ru)

Инфекциям различных видов паразитических плоских червей подвержены сотни миллионов человек по всему миру. Как острые, так и хронические инфекции в отсутствие лечения с высокой частотой приводят к развитию тяжелых патологий и даже к смерти. Данные о снижении эффективности некоторых важных противогельминтных лекарственных препаратов и развитии резистентности к ним вынуждают исследователей искать альтернативные соединения. Паразитические плоские черви обладают сложным жизненным циклом, трудоемки и дорогостоящи в разведении, а также имеют ряд приспособлений, осложняющих работу с ними стандартными молекулярно-биологическими методами. Напротив, эволюционно близкородственные паразитическим плоским червям свободноживущие виды плоских червей лишены вышеописанных трудностей, что делает их перспективными альтернативными модельными объектами для поиска и исследования гомологичных генов. В этом обзоре мы описываем применение базального свободноживущего плоского червя Macrostomum lignano в качестве такой модели. M. lignano обладает большим набором удобных биологических и экспериментальных особенностей, таких как быстрое время репродукции, дешевизна и легкость в лабораторном разведении, оптическая прозрачность тела, облигатное половое размножение, аннотированные геномные и транскриптомные сборки, а также доступность современных молекулярных методов исследования, включая трансгенез, генный нокдаун с помощью РНК-интерференции и гибридизацию in situ. Все это делает M. lignano пригодным для применения самых современных подходов «прямой» и «обратной» генетики, таких как транспозонный инсерционный мутагенез и методы направленного редактирования генома с использованием системы CRISPR/Cas9. Благодаря растущему количеству доступных сборок геномов и транскриптомов различных видов паразитических плоских червей новые знания, полученные в исследованиях на M. lignano, могут быть легко транслированы на паразитических плоских червей с применением современных биоинформационных подходов сравнительной геномики и транскриптомики. В подтверждение этому мы приводим результаты нашего биоинформационного поиска и анализа гомологичных генов M. lignano и паразитических плоских червей, которые позволили определить список перспективных генов-мишеней для дальнейшего исследования.

## Introduction 

Hundreds of millions of people worldwide are infected by various species of parasitic flatworms (Waikagul et al., 2018). The highest frequency of infections, as well the most severe pathologies, are induced by the species of the class Trematoda, or liver flukes, which cause such well-known diseases as schistosomiasis, clonorchiasis, and opisthorchiasis. Characteristic severe effects of the liver flukes infections are acute and chronic inflammation of liver and biliary tract, which can develop into liver fibrosis and cholangiocarcinoma, respectively (Wongratanacheewin et al., 2003; Kaewpitoon et al., 2008; Andrade, 2009; Pomaznoy et al., 2016; Schwartz, Fallon, 2018). Infections of another class of parasitic flatworms, Cestoda, or tape worms, often do not lead to such severe pathologies and death, but in the long-term perspective and without treatment they can lead to significant aberrations in vital activity and as a consequence a decrease in life quality of sick people (Budke et al., 2009; Waikagul et al., 2018).

In the world, for more than 40 years praziquantel and its derivatives have been the “number one” drugs against helminthiases (Chai, 2013; Pakharukova et al., 2015). However, continuous and widespread use of praziquantel has already resulted in the increasing number of reports on emerging resistance to the drug in different species of helminthes (Botros, Bennett, 2007; Wang et al., 2012; Mwangi et al., 2014; Jesudoss Chelladurai et al., 2018). An induced resistance to praziquantel was experimentally demonstrated in some schistosomes (Mwangi et al., 2014). Inital successes of praziquantel slowed down investments into the development of new anthelmintic drugs, which further complicates the situation. At the same time, the developed alternatives to praziquantel demonstrate analogous or sometimes even lower efficiency, more side effects, and usually are effective only against certain trematode species (Siqueira et al., 2017). Therefore, there is an urgent need for new and more effective anthelmintic drugs

Parasitic flatworms have complex life cycles with several changes of the hosts (Morand et al., 1995; Poulin, Cribb, 2002), are laborious and expensive in laboratory culturing, and have numerous specific adaptations that complicate their study by standard molecular techniques. All these properties, undoubtedly, slow down fast development of new anthelmintic drugs. Our knowledge on a broad spectrum of biological questions was gained via research on convenient model organisms, such as nematodes, fruit flies, mice, yeast, etc. Similarly, studies of free-living animals help to obtain new information about their parasitic relatives. For example, investigating model free-living roundworm (nematode) Caenorhabditis elegans, new data were obtained, which allowed description of a more detailed mechanism of action for some anti-nematode drugs, as well as helped the search for new genes potentially regulating the life cycle of parasitic nematodes. Subsequently, these genes can be used as targets for developing new drugs (Cully et al., 1994; Couthier et al., 2004; Guest et al., 2007; Laing et al., 2010). Among flatworms, free-living species can be used as models to screen for new drugs directed against their parasitic relatives (Collins, Newmark, 2013). Despite fundamental differences in the life cycles, free-living flatworms have a set of evolutionary conserved properties of their physiology and reproduction, which are shared with parasitic species.

In this study, we describe the properties, advantages, and potential application of the free-living flatworm Macrostomum lignano as a convenient research model for efficient screening of conserved genes homologous to the genes of parasitic flatworms, which can serve as targets for the development of new anthelmintic drugs. 

## General properties of Macrostomum lignano as a model

Macrostomum lignano is a free-living flatworm (phylum Platyhelminthes, class Rhabditophora) from a basal (the earliest branching) clade – Macrostomorpha (Ladurner et al., 2005; Egger et al., 2015). M. lignano can easily tolerate a wide range of different environmental conditions, such as temperature, salinity, and oxygen concentration (RiveraIngraham et al., 2013, 2016; Wudarski et al., 2019). It was experimentally demonstrated that the worms can survive at the temperatures between 4 to 37 °C (Wudarski et al., 2019). M. lignano is easy to culture in laboratory conditions (Wudarski et al., 2020). The size of adult animals varies from 1 to 3 mm in length and 0.3 mm in width. Worms are maintained in Petri dishes with artificial sea water. A species of unicellular diatom algae Nitzschia curvilineata, which is itself easy to culture in laboratory conditions under artificial illumination, is used as food source. In one standard (9 cm) Petri dish, 500–600 individuals can be easily simultaneously maintained. Standard cultivation temperatures are 20 °C and 14/10 hours day/night light cycle.

Free-living flatworms are famous for their high regeneration capacity (Egger et al., 2006; Mouton et al., 2018; Ivankovic et al., 2019). The known champions are planarians, which can restore a full-grown animal from just a few cells (Wagner et al., 2011). M. lignano is nearly as regenerative as planarians, and can fully regenerate its body posterior from the pharynx and anterior to the brain (Egger et al., 2006). Flatworm regeneration comes from division and differentiation of somatic stem cell population called neoblasts (Wagner et al., 2011). Neoblasts and their differentiating progenitors are the only dividing cells in flatworms, and, apart from regeneration, they are also responsible for the natural tissue renewal during homeostasis (Nimeth et al., 2002; Ladurner et al., 2008). Importantly, there are also neoblast-like cells in parasitic flatworms, which are morphologically similar to neoblasts described in free-living species (Brehm, 2010; Collins, Newmark, 2013; Collins et al., 2013; McCusker et al., 2016). Neoblast-like cells can differentiate into other cell types and are responsible for regeneration of lost body parts in parasitic flatworms, as well as have similar transcriptional profiles to neoblasts from free-living species. Thus, there is an obvious homology of central systems of homeostasis and regeneration between free-living and parasitic flatworms.

An important advantage of M. lignano compared to other popular free-living model flatworms – planarians – is its body transparency (Ivankovic et al., 2019; Wudarski et al., 2020). This substantially facilitates morphological studies of its internal structures with the help of light microscopy. M. lignano is an obligatory reciprocal hermaphrodite, favorably distinguishing it from planarians, which in laboratory conditions reproduce predominantly asexually through fission, and are also genetically mosaic even within an individual (Schärer, Ladurner, 2003; Leria et al., 2019). Obligatory sexual reproduction of M. lignano allows its application in controlled genetic studies

Currently, the presence of a simple and efficient method for transgenesis is the unique feature of M. lignano among other flatworm species (Wudarski et al., 2017). M. lignano lays 1–2 single cell eggs per day. Eggs are large (~100 μm), have relatively hard shells, and can be easily manipulated with the help of plastic microtools. These properties allowed the development of a successful protocol for delivery of various genetic constructs (DNA, mRNA, proteins) inside the eggs by means of microinjection (Wudarski et al., 2017, 2020). To date, there is a range of M. lignano transgenic lines which express genes of reporter green and red fluorescent proteins in different organs and tissues, allowing to study the place and dynamics of expression of a gene of interest in vivo (Wudarski et al., 2017, 2019). 

Apart from transgenesis, other classical molecular and cytological methods are successfully applied in M. lignano. Localization of a gene of interest expression can be studied by means of in situ hybridization (Pfister et al., 2007; Grudniewska et al., 2016; Wudarski et al., 2017; Lengerer et al., 2018). To identify gene function, there is a very simple and efficient protocol for knockdown of gene expression by RNA interference, and there is no need for special delivery of double-stranded (dsRNA) constructs – worms are simply soaked in dsRNA solution and after 1–3 weeks, due to the transparency of M. lignano, it is possible to observe occurred morphological, physiological, or behavior changes (Grudniewska et al., 2016, 2018; Lengerer et al., 2018; Wudarski et al., 2019). Thus, the available experimental methods allow implementation of complex studies on the expression and gene function in M. lignano.

Any modern model organism needs a well-assembled genome and transcriptome assembly with annotation of genes and repetitive sequences, transposons and simple/ tandem repeats. M. lignano is not an exception (Wasik et al., 2015; Grudniewska et al., 2016, 2018; Wudarski et al., 2017; Biryukov et al., 2020). M. lignano has a relatively compact genome of ~500 Mb. Genome and transcriptome assemblies can be openly accessed and viewed using the convenient web-interface http://gb.macgenome.org/ (Wudarski et al., 2017; Grudniewska et al., 2018). We already know genes that are differentially expressed specifically in neoblasts and the worm germline (Grudniewska et al., 2016, 2018). Thus, M. lignano can be used for computational analysis of evolution, comparative genomics and transcriptomics to search for conserved genes homologous to parasitic flatworms. Main properties of M. lignano, planarians, and parasitic flatworms are summarized in the Table.

**Table 1.**
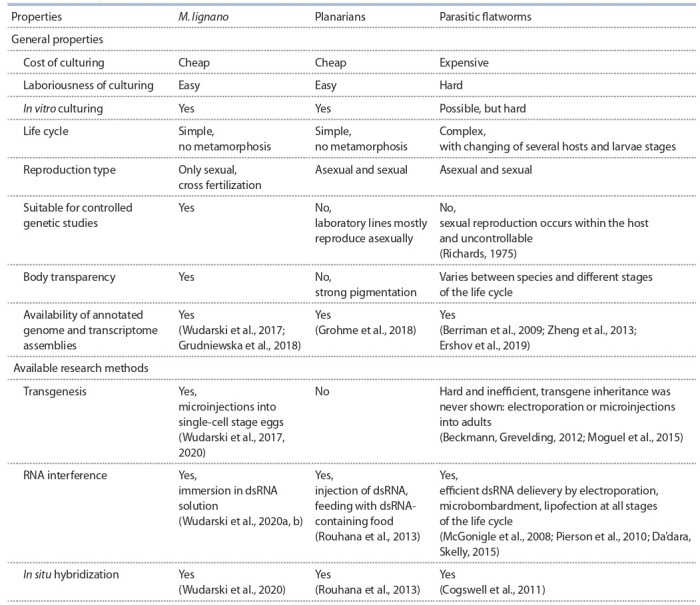
Comparison of key properties of free-living flatworms M. lignano and planarians, and parasitic flatworms as model organisms

## Specific features of M. lignano as a model to search for gene targets regulating germline development and function in parasitic flatworms

Development of acute and chronic inflammation is an important hallmark of trematode-caused pathologies, which are caused by constant egg laying of the parasites, leading to the activation of the immunological response, which is especially relevant to schistosomiasis (Wongratanacheewin et al., 2003; Kaewpitoon et al., 2008; Collins, Newmark, 2013; Schwartz, Fallon, 2018). Thus, the germline of helminths and genes that control its development and homeostasis appear as promising targets for the development of new drugs directed to suppress their expression. 

In a recent work on M. lignano (Grudniewska et al., 2018) it was shown that the majority of its genes classified as germline-specific are flatworm-specific (both for free-living and parasitic species) and lack a homolog in human and other model organisms. Investigation of flatworm-specific genes can be the key to search for new anthelmintic drugs with fewer side effects due to their target action on the gene products absent in humans. M. lignano is a convenient model to screen for such targets. As mentioned earlier, all organs of its reproductive system are clearly distinguishable under a common light dissecting microscope. This significantly facilitates the screening of phenotypes linked to the disruption of genes active in gonads and/or copulative organs (Grudniewska et al., 2018). Importantly, the worm hermaphroditism will allow maintaining in populations genetic aberrations linked to the activity of either male or female reproductive systems. Disturbances in fertility will already be detectable within a week at 25 °C (Wudarski et al., 2019), which will help not to miss mutations in the absence of a clear morphological phenotype.

## Main methods and application of M. lignano for comparative genomics

Now we are already at the beginning of the era of targeted genome editing that started with the wide spread of CRISPR/Cas9 technology (Anzalone et al., 2020). Given a well-annotated genome assembly, it is possible to introduce mutations to a certain gene of interest, which would lead to complete disruption of its function (knockout) (Chen et al., 2014). Of particular interest is insertion of marker reporter sequences (e. g. fluorescent proteins) directly in the open reading frame of a target gene (knockin), which allows direct visualization of the gene expression pattern by the localization of the encoded protein (Albadri et al., 2017; Artegiani et al., 2020). For example, by combining labeling of several proteins by different fluorescent proteins, interactome studies are possible. 

The function of CRISPR/Cas9 depends on only two (in the case of knockouts) or three (in the case of knockins) components: guide RNA, Cas9 nuclease protein, and a matrix for homologous recombination. In the simplest scenario, these are two plasmid vectors, one of which encodes guide RNA and Cas9, and the other is the matrix for homologous recombination (Hsu et al., 2014). Alternatively, this can be a combination of in vitro synthesized guide RNA and Cas9 in the form of mRNA or Cas9 protein in the complex with the guide RNA, which eliminates the possibility for unwanted insertion of the plasmid vector (Hsu et al., 2014; Kim et al., 2014). Successful and reproducible application of CRISPR/Cas9 is impossible without an efficient delivery of genetic constructs (DNA, mRNA or proteins). Currently, M. lignano is the only flatworm for which this is possible by means of microinjection into single-cell stage eggs of the worm (Wudarski et al., 2017). Such an approach is certainly the most effective, since all the components of the systems are delivered simultaneously in the required molar ratio at the single-cell stage, which decreases chances for mosaic progeny. Although currently there are no published data on the application of CRISPR/Cas9 in M. lignano, our preliminary experiments show that this approach can be efficiently applied for a knockin introduction in the M. lignano genome.

Studies of phenotypes after targeted disruption/labeling of a gene of interest are characteristic of reverse genetics methods (Pareek et al., 2018). The main disadvantage of this approach is that a high-quality assembly and the annotation of the genome are required for the correct selection of the modification site and the preliminary assessment of the gene function based on its homology to already known proteins (Skromne, Prince, 2008). Moreover, genome editing by CRISPR/Cas9 depends on how frequently a GG pattern occurs in the genome, as the Cas9 protein must first detect a PAM-site (Protospacer Adjacent Motif) NGG in the target sequence (Hsu et al., 2014). An additional problem is that different guide RNAs vary significantly in their efficiency of double-strand break induction, and it is rarely possible to exactly predict the efficiency during the in silico design (Chuai et al., 2017). While classical models, such as human cell lines, mouse, Drosophila, the nematode C. elegans, and yeasts are thoroughly studied and there are enough data on their gene function to predict a phenotype, and their genomic GC-content is optimal, the situation with alternative models is different.

The function of a gene is rarely known, as it can be conserved only within a certain evolutionary taxon (e.g. the case of flatworm germline-specific genes). The genome can have a low GC-content, less than 40 %, which lowers the probability to meet a GG in the target regions that could be mutated to result in the target gene knockout (Casandra et al., 2018). In such cases, one should follow a historically earlier approach of forward genetics: from a phenotype to the gene (Pareek et al., 2018). 

Transposon insertional mutagenesis is the most developed tool among the methods of forward genetics. Compared to chemical mutagens, which induce mutations throughout the genome but require significant time to map the mutation, a transposon movement and its insertion place can be easily detected by modern methods within one-two days (Potter, Luo, 2010; Frøkjær-Jensen et al., 2012; Stefano et al., 2016; Kalendar et al., 2019). This is achieved because the transposon sequence is originally not present in the studied genome; various promoters, enhancers, and gene trapping reporter constructs can be put in the transposon to additionally report on its insertion as well (Bonin, Mann, 2004; Song et al., 2012; Chang et al., 2019). In a recent study on the malaria parasite, it was transposon mutagenesis using the piggyBac DNA transposon that allowed to create 38,000 mutants of the plasmodium, and in these mutants 2680 genes regulating the parasite reproduction in blood cells were identified (Casandra et al., 2018). The authors note that it was not possible to apply CRISPR/Cas9 due to anomalously low GC-content (< 20 %) of the plasmodium genome. M. lignano and other flatworms, including parasitic ones, are now far from being classical and ubiquitously used model objects. As mentioned above, genes specific to the germline of flatworms mostly lack a homolog in other animals, eliminating the predictive power of the reverse genetics methods. Thus, transposon mutagenesis appears to be the most promising approach to search for the genes regulating flatworm germline, as well as other flatworm-specific genes controlling other functions, and the development of an efficient protocol for transposon mutagenesis in M. lignano is warranted

Importantly, new knowledge gained from experiments on M. lignano can be transferred to parasitic flatworms due to availability of numerous assemblies of genomes and transcriptomes for the most significant parasitic species, which are accessible at the WormBase ParaSite (https://parasite. wormbase.org/index.html) database (Berriman et al., 2009; Zheng et al., 2013; Cwiklinski et al., 2015; Ershov et al., 2019). By using modern computational tools of comparative genomics and transcriptomics, it is possible to readily identify the sequences of potential target genes revealed in M. lignano, which are homologous in different parasitic flatworm species, and to perform their comparative and phylogenetic analyses in silico. This will allow to select candidate genes that will be the most conserved throughout all parasitic flatworm genomes, and (preferably) have weak homology to human genes.

## Computational analysis of conserved genes between M. lignano and parasitic flatworms

From the WormBase ParaSite database, amino acid sequences of protein-coding genes from 31 parasitic flatworm species were retrieved: 14 species from the class Trematoda, 15 species from Cestoda, and 2 species from Monogenea (see Figure, a, Supplementary 1)^1^.

^1^ Supplementary materials 1–2 are available in the online version of the paper:http://www.macgenome.org/download/pdf/Ustyantsev_2021/


**Fig. 1.**
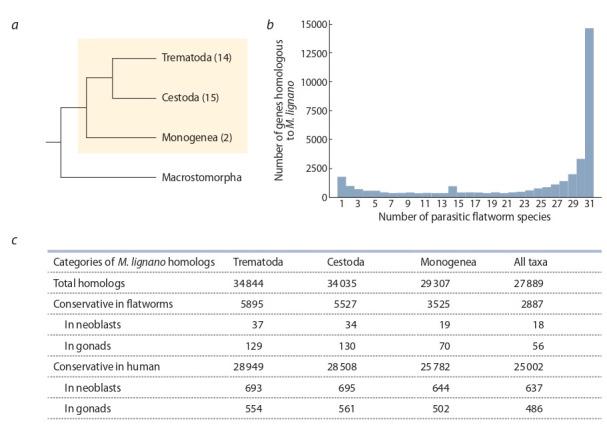
Homology of genes in M. lignano and parasitic flatworm species a – phylogenetic relationships between M. lignano (Macrostomorpha) and parasitic flatworm classes according to (Park et al., 2007). Number of species in WormBase ParaSite database used in the analysis is shown in parentheses next to the taxa names; b – distribution of homologous genes among the number of the studied parasitic flatworm species; c – distribution of M. lignano homologous genes among parasitic flatworm classes. Number of homologs found at least in one species of each class is shown in the “All taxa” column

## Conclusion

In this study, we highlighted the key properties of free-living flatworm M. lignano as a model organism, and those that make it a promising object for fast and efficient screening of potential anthelmintic drugs. The availability of easy to implement transgenesis in M. lignano opens access to the whole arsenal of the modern methods in molecular biology to study gene functions, and its body transparency allows in vivo monitoring of phenotypical changes caused by gene disruption or labeling by methods of forward and reverse genetics without additional manipulations. Genes regulating development and germline functioning in flatworms appear as the most promising targets, since they are conserved among flatworms and have no homologs in human.

## Conflict of interest

The authors declare no conflict of interest.
